# Supplementary material for: Mapping the Constrained Coding Regions in the Human Genome to Their Corresponding Proteins
Source: J Mol Biol. 2023 Jan 30;435(2):167892. doi: 10.1016/j.jmb.2022.167892 (PMC9875310; doi:10.1016/j.jmb.2022.167892)
Supplement: Supplementary Figures — Supplementary Figure 1: comparison of pLI and missense OEUF and the maximum CCRs percentile by protein. Supplementary Figure 2: The higher the constrained percentile the larger the mean length of the regions and the higher the mean conservation of the amino acids within them. However, each percentile category exhibits a high variability. Supplementary Figure 3: Comparison of full length of proteins and the number of regions in each CCRs percentile that they have. Supplementary Figure 4: Distribution of the raw count of amino acid positions grouped by different categories of CCRs percentiles and conservation scores. Supplementary figure 5: Comparison of OR tests, conservation and length of regions by protein feature. Supplementary figure 6: Comparison of OR tests, conservation and length of regions by type of ClinVar variants. Supplementary Figure 7: Amino acid positions affected with clinically interpreted variants assessed by their location in the different protein features or functional sites. [file mmc1.pdf]

Mapping the Constrained Coding Regions in the human genome to their corresponding proteins

Marcia A. Hasenahuer[1,2,5], Alba Sanchis-Juan[3,4,6], Roman A. Laskowski[1], James A. Baker[1], James D. Stephenson[1], Christine A. Orengo[5], F. Lucy Raymond[2,4], Janet M. Thornton[1]

Supplementary Figures

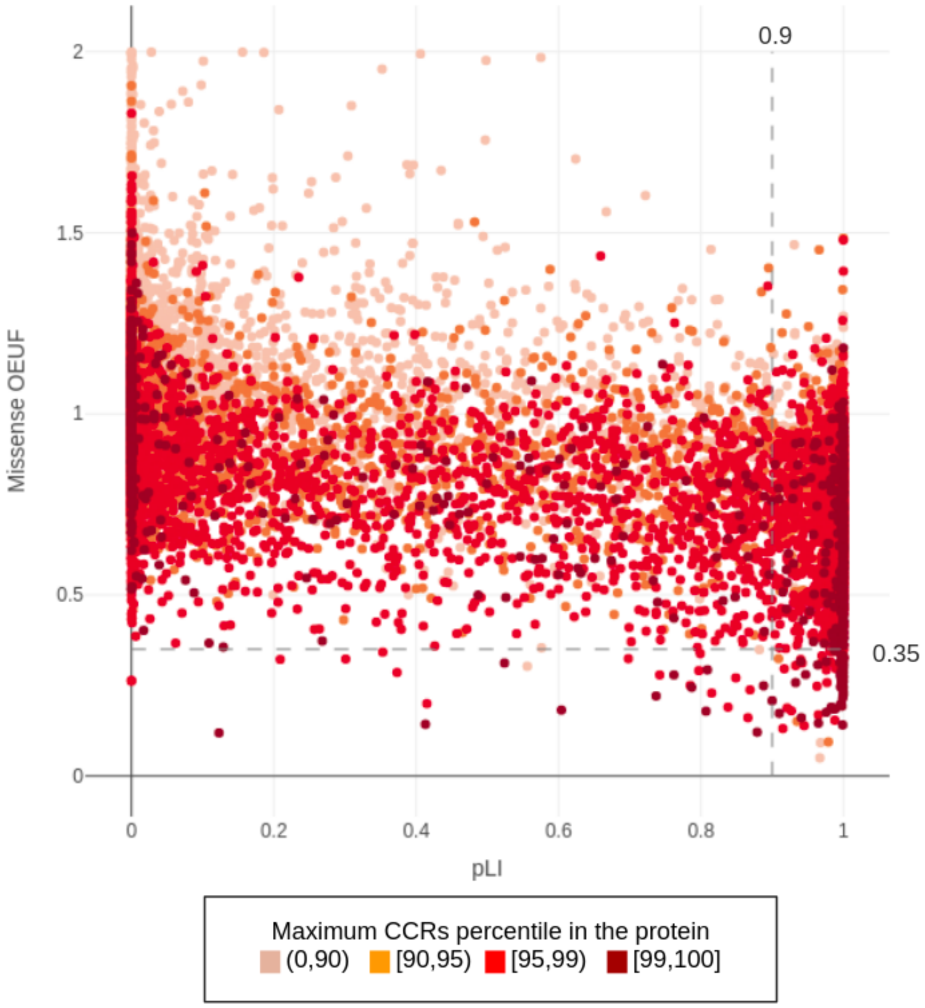

Supplementary figure 1: Comparison of pLI and missense OEUF and the maximum CCRs percentile by protein. Thresholds for missense OEUF (≤0.35) and pLI (≥0.9) scores, showing the proteins (dots) with the highest constraints for missense and pLoF respectively, are depicted with dashed lines. The lower right corner, enclosed by both thresholds, groups genes highly constrained for pLoF and missense variants

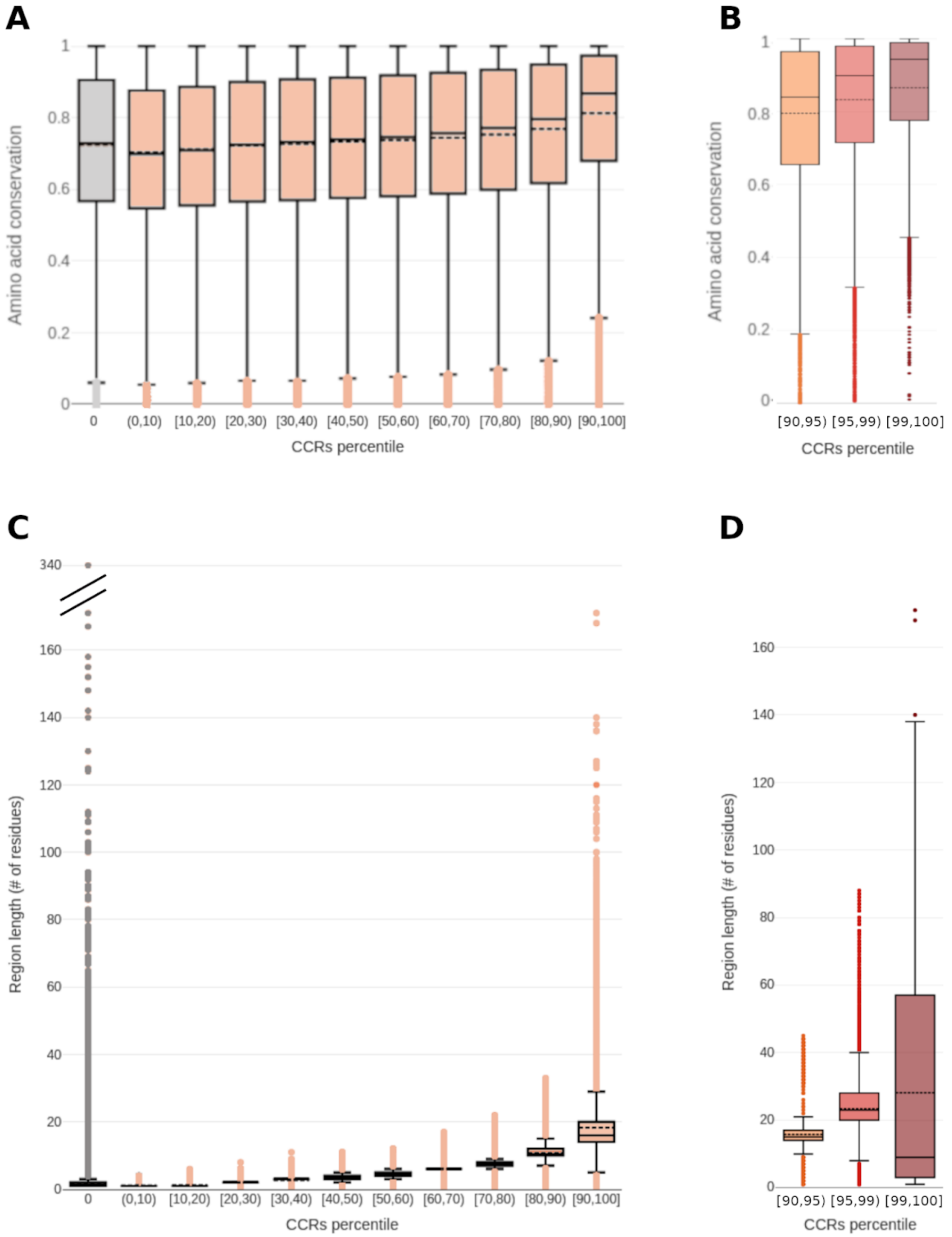

Supplementary figure 2: The higher the constrained percentile the larger the mean length of the regions and the higher the mean conservation of the amino acids within them. However, each percentile category exhibits a high variability. The boxplots in the upper panels, (A) and (B), show the distributions of amino acid conservation for all the mapped positions. In the lower panels, (C) and (D), the distribution of lengths for all the mapped regions are shown. Left panels (A) and (C) show all the CCRpct for unconstrained (0 pct) and constrained categories, sub-categorised into intervals of ten. The right panels, (B) and (D), focus only on the distribution of the subset of medium to highly constrained positions and regions, i.e. percentiles [90,100], subdivided into three categories from medium to most highly constrained. The colouring follows the same criteria as described in Figure 1 B.

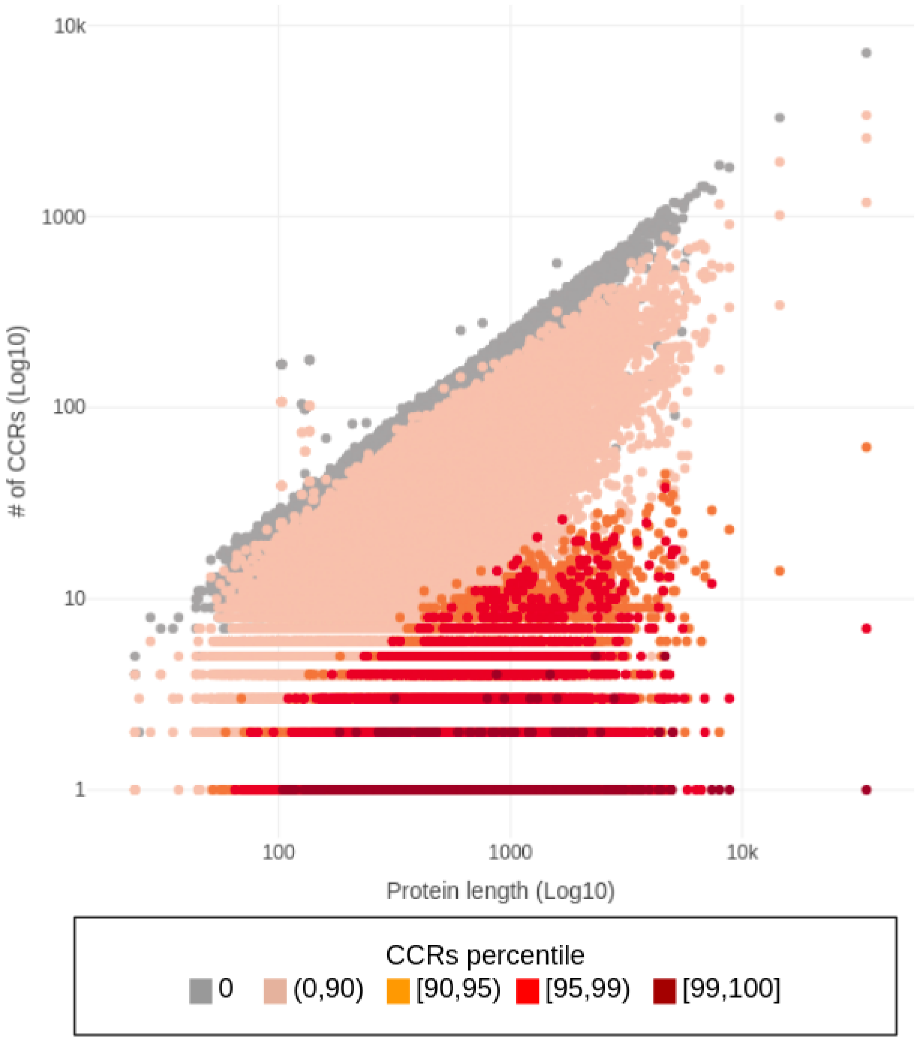

Supplementary Figure 3: Comparison of full length of proteins and the number of regions in each CCRs percentile that they have.

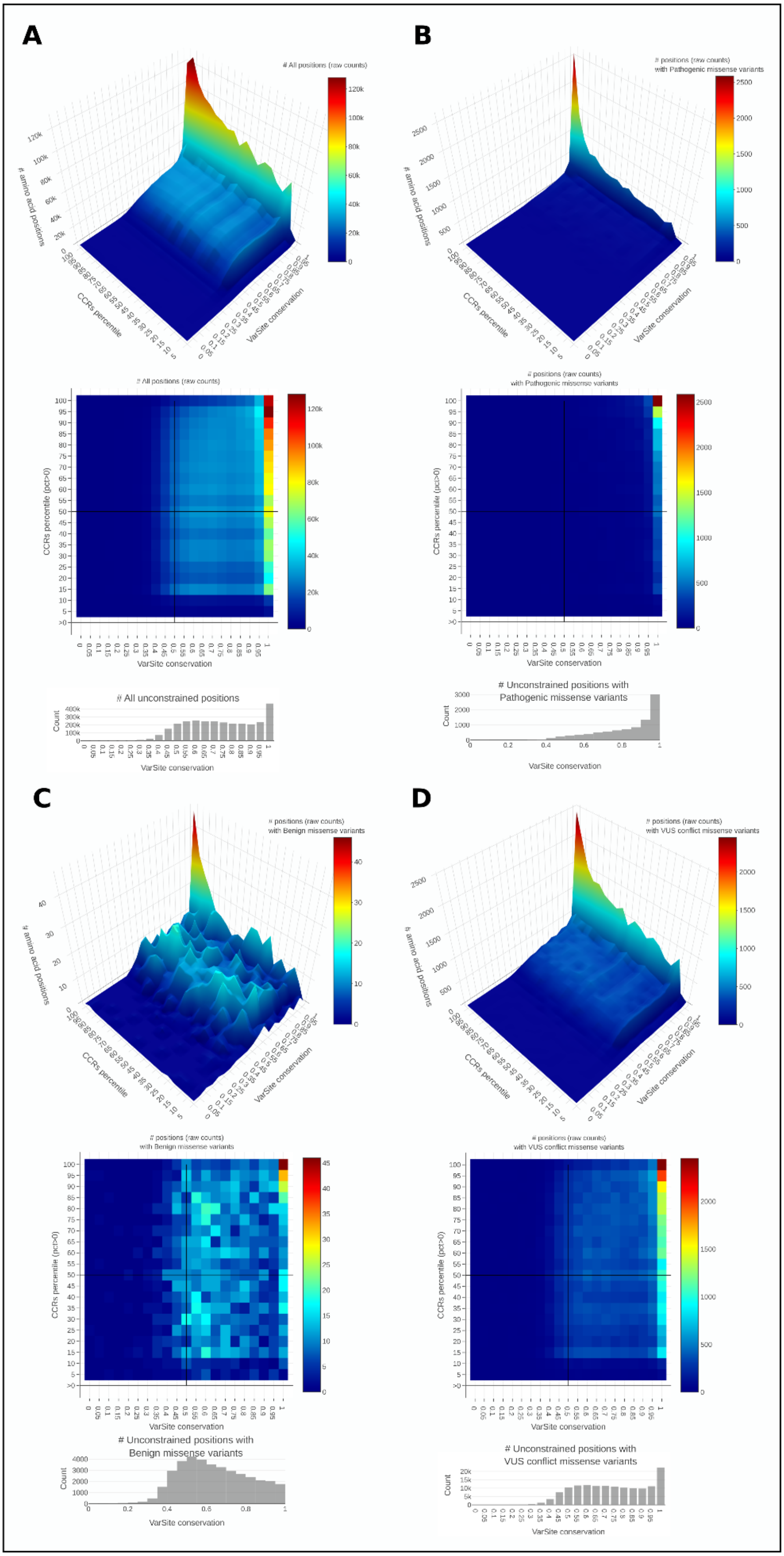
A
All positions (raw counts)
amino acid positions
CCRs percentile
VarSite conservation
CCRs percentile (pct>0)
All unconstrained positions
Count
B
positions (raw counts)
with Pathogenic missense variants
Unconstrained positions with
Pathogenic missense variants
C
positions (raw counts)
with Benign missense variants
Unconstrained positions with
Benign missense variants
D
positions (raw counts)
with VUS conflict missense variants
Unconstrained positions with
VUS conflict missense variants

Supplementary Figure 4: Distribution of the raw count of amino acid positions grouped by different categories of CCRs percentiles and conservation scores. A) all positions, and those affected by B) Pathogenic/Likely_pathogenic, C) Benign/Likely_benign and D) VUS/conflictive variants.

in DOMAIN

CCRs pct	[T][T]	[T][F]	[F][T]	[F][F]	OR	CI 95%	Fisher p-value
0,0]	1065616	2618209	2003314	4139901	0.84	0.84-0.84	0 *
0,30)	483897	3199928	915102	5227648	0.86	0.86-0.87	0 *
30,60)	803415	2880410	1414811	4728123	0.93	0.93-0.94	0 *
60,90)	961599	2722226	1450553	4692355	1.14	1.14-1.15	0 *
90,95)	198797	3485028	226160	5916047	1.49	1.48-1.5	0 *
95,99)	157074	3526751	125600	6016533	2.13	2.12-2.15	0 *
99,100]	15221	3668604	8386	6133688	3.03	2.95-3.12	0 *

in TRANSMEMBRANE

CCRs pct	[T][T]	[T][F]	[F][T]	[F][F]	OR	CI 95%	Fisher p-value
[0,0]	116477	272003	2952453	6486863	0.94	0.93-0.95	8.0e-66 *
(0,30)	52412	336068	1346586	8091985	0.94	0.93-0.95	2.1e-42 *
[30,60)	88398	300082	2129825	7309086	1.01	1-1.02	5.4e-03 *
[60,90)	100808	287672	2311342	7127536	1.08	1.07-1.09	1.6e-94 *
[90,95)	17748	370732	407209	9030502	1.06	1.05-1.08	4.0e-14 *
[95,99)	11883	376597	270791	9166717	1.07	1.05-1.09	6.8e-12 *
[99,100]	881	387599	22726	9414687	0.94	0.88-1.01	8.2e-02

in DISORDER-FLEXIBLE

CCRs pct	[T][T]	[T][F]	[F][T]	[F][F]	OR	CI 95%	Fisher p-value
[0,0]	619721	1192921	2449209	5565293	1.18	1.18-1.18	0.0e+00 *
(0,30)	281918	1530724	1117080	6896904	1.14	1.13-1.14	0.0e+00 *
[30,60)	405809	1406833	1812414	6201835	0.99	0.98-0.99	3.8e-11 *
[60,90)	395676	1416966	2016474	5997809	0.83	0.83-0.83	0.0e+00 *
[90,95)	62563	1750079	362394	7651042	0.75	0.75-0.76	0.0e+00 *
[95,99)	43232	1769410	239442	7773865	0.79	0.79-0.8	0.0e+00 *
[99,100]	5051	1807591	18556	7994695	1.2	1.17-1.24	1.8e-30 *

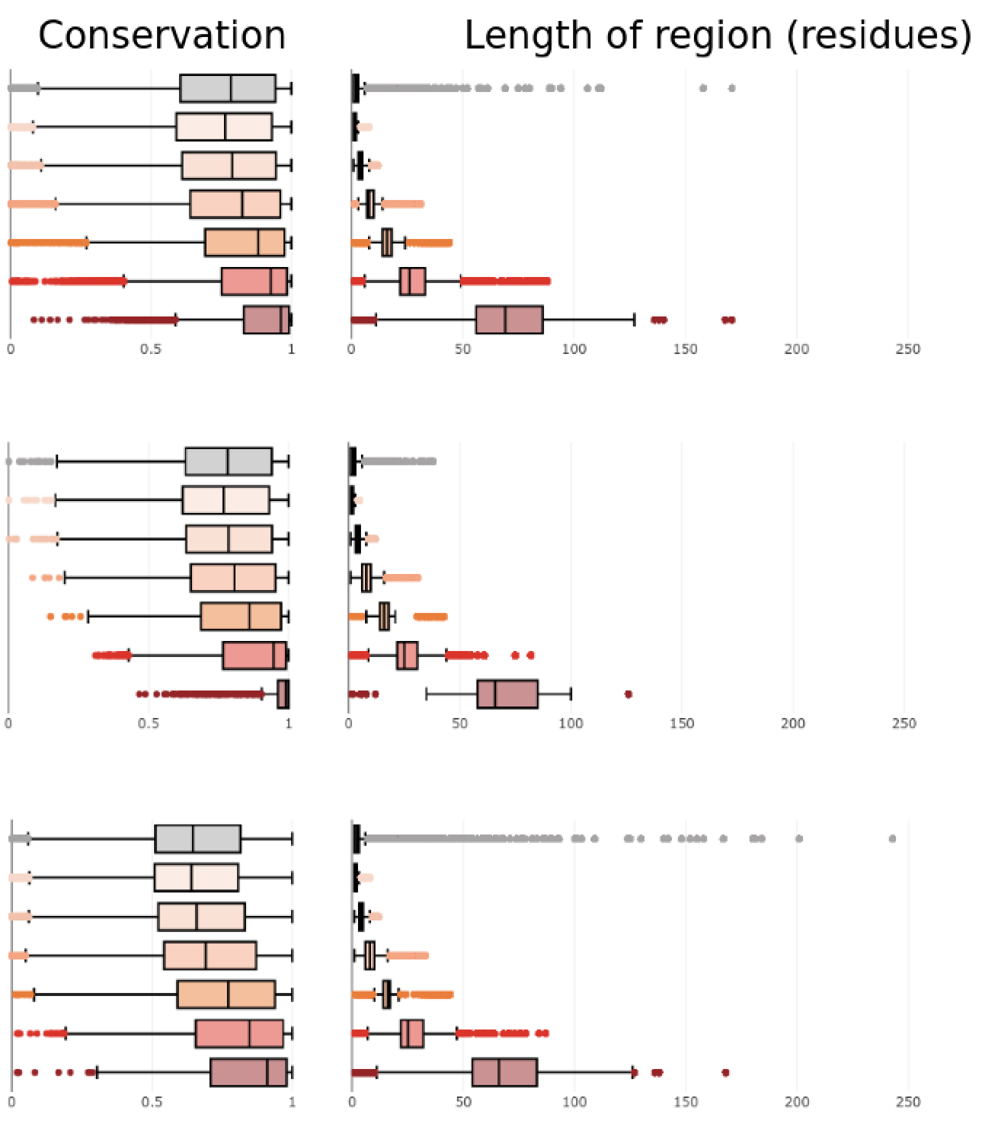

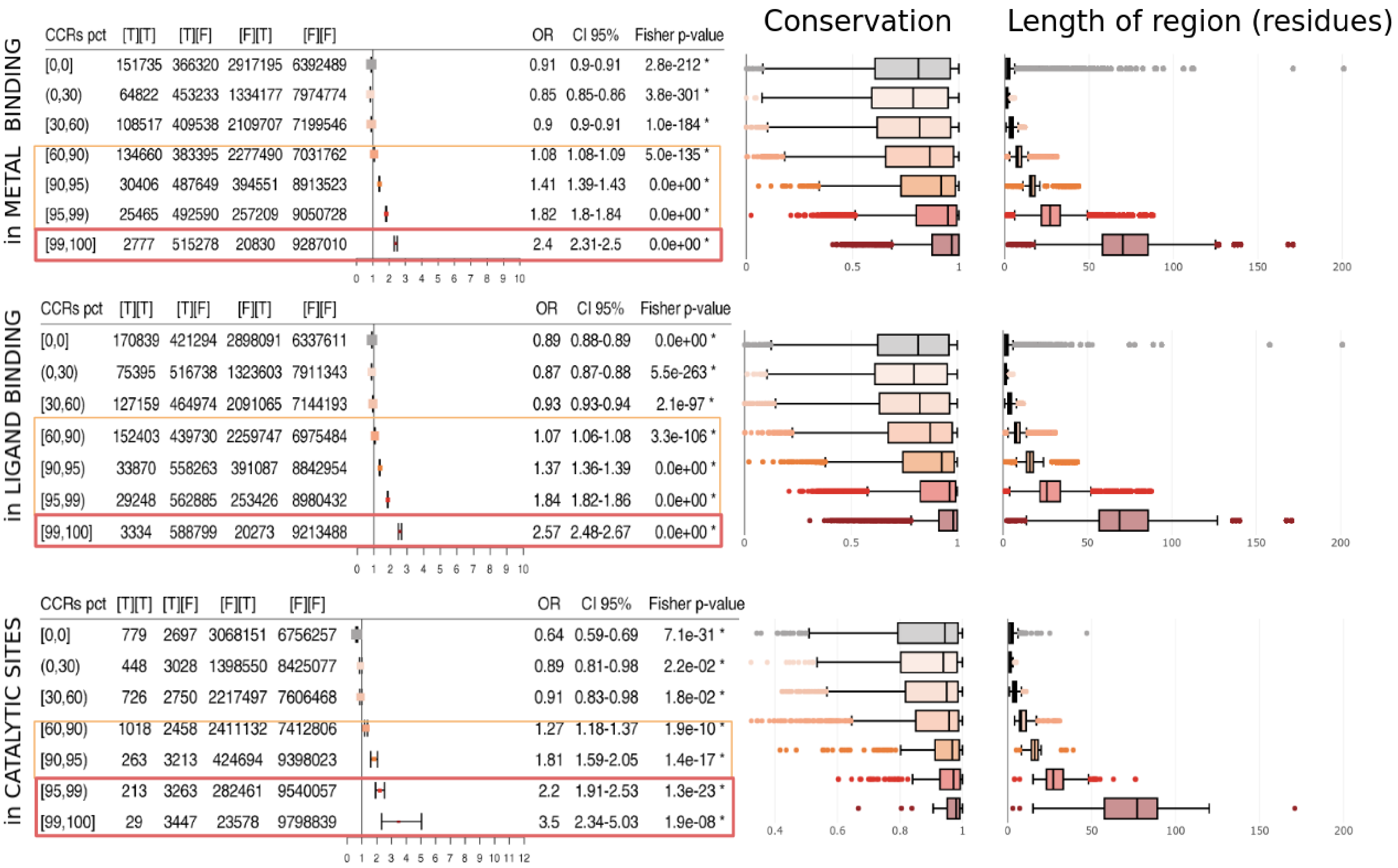

in METAL BINDING

CCRs pct	[T][T]	[T][F]	[F][T]	[F][F]	OR	CI 95%	Fisher p-value
[0,0]	151735	366320	2917195	6392489	0.91	0.9-0.91	2.8e-212 *
(0,30)	64822	453233	1334177	7974774	0.85	0.85-0.86	3.8e-301 *
[30,60)	108517	409538	2109707	7199546	0.9	0.9-0.91	1.0e-184 *
[60,90)	134660	383395	2277490	7031762	1.08	1.08-1.09	5.0e-135 *
[90,95)	30406	487649	394551	8913523	1.41	1.39-1.43	0.0e+00 *
[95,99)	25465	492590	257209	9050728	1.82	1.8-1.84	0.0e+00 *
[99,100]	2777	515278	20830	9287010	2.4	2.31-2.5	0.0e+00 *

in LIGAND BINDING

CCRs pct	[T][T]	[T][F]	[F][T]	[F][F]	OR	CI 95%	Fisher p-value
[0,0]	170839	421294	2898091	6337611	0.89	0.88-0.89	0.0e+00 *
(0,30)	75395	516738	1323603	7911343	0.87	0.87-0.88	5.5e-263 *
[30,60)	127159	464974	2091065	7144193	0.93	0.93-0.94	2.1e-97 *
[60,90)	152403	439730	2259747	6975484	1.07	1.06-1.08	3.3e-106 *
[90,95)	33870	558263	391087	8842954	1.37	1.36-1.39	0.0e+00 *
[95,99)	29248	562885	253426	8980432	1.84	1.82-1.86	0.0e+00 *
[99,100]	3334	588799	20273	9213488	2.57	2.48-2.67	0.0e+00 *

in CATALYTIC SITES

CCRs pct	[T][T]	[T][F]	[F][T]	[F][F]	OR	CI 95%	Fisher p-value
[0,0]	779	2697	3068151	6756257	0.64	0.59-0.69	7.1e-31 *
(0,30)	448	3028	1398550	8425077	0.89	0.81-0.98	2.2e-02 *
[30,60)	726	2750	2217497	7606468	0.91	0.83-0.98	1.8e-02 *
[60,90)	1018	2458	2411132	7412806	1.27	1.18-1.37	1.9e-10 *
[90,95)	263	3213	424694	9398023	1.81	1.59-2.05	1.4e-17 *
[95,99)	213	3263	282461	9540057	2.2	1.91-2.53	1.3e-23 *
[99,100]	29	3447	23578	9798839	3.5	2.34-5.03	1.9e-08 *

DISORDER-to-ORDER transition

CCRs pct	[T][T]	[T][F]	[F][T]	[F][F]	OR	CI 95%	Fisher p-value
[0,0]	16258	43037	3052672	6715810	0.83	0.82-0.85	1.2e-91 *
(0,30)	7218	52077	1391780	8375950	0.83	0.81-0.86	4.4e-49 *
[30,60)	11626	47669	2206597	7561492	0.84	0.82-0.85	2.4e-69 *
[60,90)	16222	43073	2395928	7372123	1.16	1.14-1.18	4.9e-56 *
[90,95)	3699	55596	421258	9345633	1.48	1.43-1.53	1.4e-103 *
[95,99)	3980	55315	278694	9487994	2.45	2.37-2.53	0.0e+00 *
[99,100]	485	58810	23122	9743476	3.48	3.17-3.8	4.0e-113 *

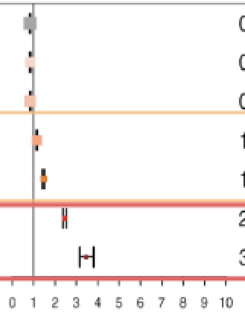

DISORDER-to-DISORDER transition

CCRs pct	[T][T]	[T][F]	[F][T]	[F][F]	OR	CI 95%	Fisher p-value
[0,0]	59277	128995	3009653	6629854	1.01	1-1.02	1.5e-02 *
(0,30)	26981	161291	1372017	8266736	1.01	0.99-1.02	2.4e-01
[30,60)	40965	147307	2177258	7461866	0.95	0.94-0.96	1.1e-17 *
[60,90)	45171	143101	2366979	7272090	0.97	0.96-0.98	1.7e-08 *
[90,95)	8376	179896	416581	9221331	1.03	1.01-1.05	7.7e-03 *
[95,99)	6850	181422	275824	9361885	1.28	1.25-1.31	3.4e-82 *
[99,100]	822	187450	22785	9614836	1.85	1.72-1.98	3.1e-56 *

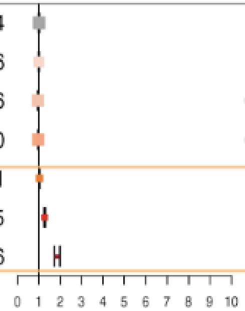

CONTEXT DEPENDENT transition

CCRs pct	[T][T]	[T][F]	[F][T]	[F][F]	OR	CI 95%	Fisher p-value
[0,0]	238	621	3068692	6758333	0.84	0.72-0.98	2.7e-02 *
(0,30)	88	771	1398910	8427334	0.69	0.55-0.86	6.1e-04 *
[30,60)	217	642	2218006	7608576	1.16	0.99-1.35	6.1e-02
[60,90)	246	613	2411904	7414651	1.23	1.06-1.43	6.2e-03 *
[90,95)	16	843	424941	9400393	0.42	0.24-0.69	1.1e-04 *
[95,99)	46	813	282628	9542507	1.91	1.39-2.57	8.6e-05 *
[99,100]	8	851	23599	9801435	3.9	1.68-7.73	1.3e-03 *

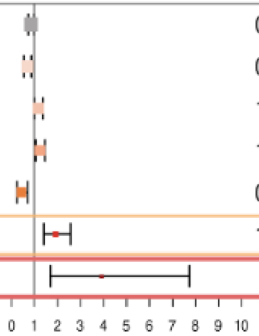

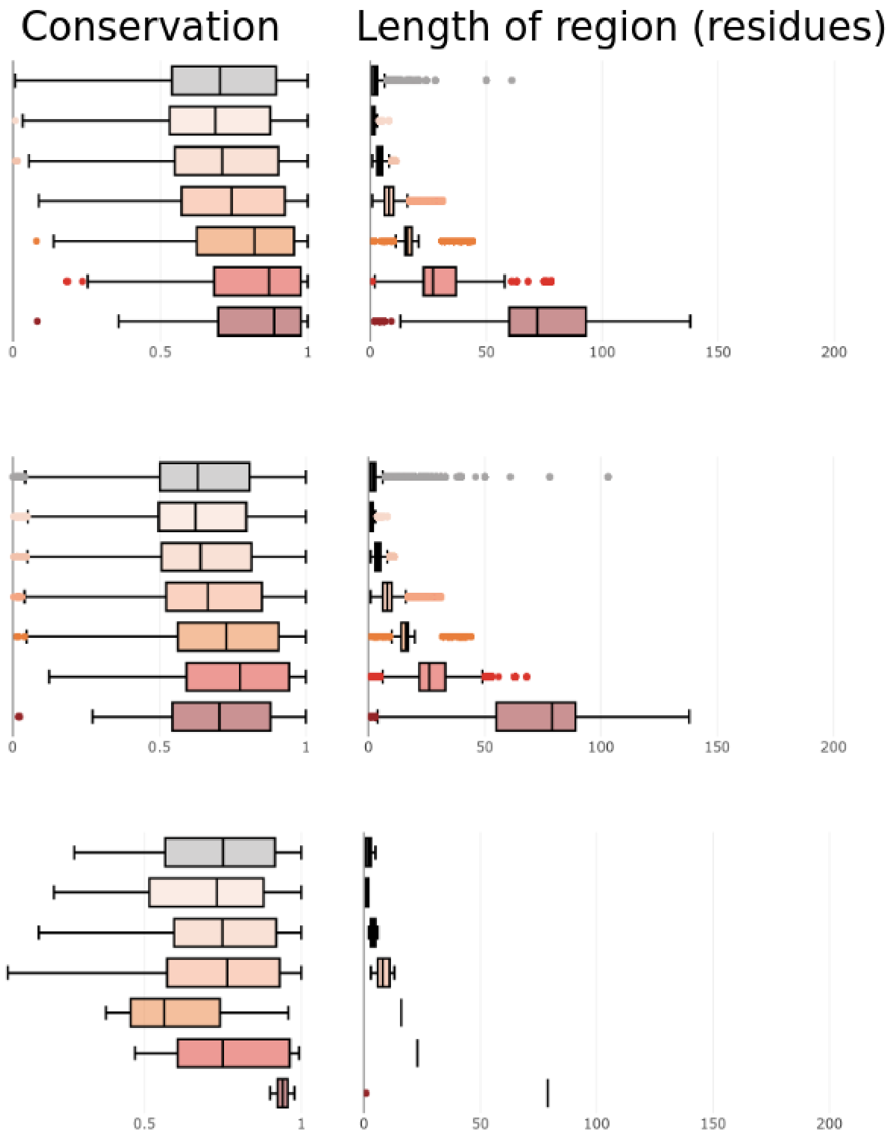

Supplementary figure 5: Comparison of OR tests, conservation and length of regions for selected protein features. For each protein feature or functional site, the plots show: the contingency tables with the sites counts, OR, 95% CI and p-values along with boxplots of distribution of CCRs regions length and interspecies amino acid conservation. Stars (*) highlight statistically significant ORs (p-value ≤ 0.05)

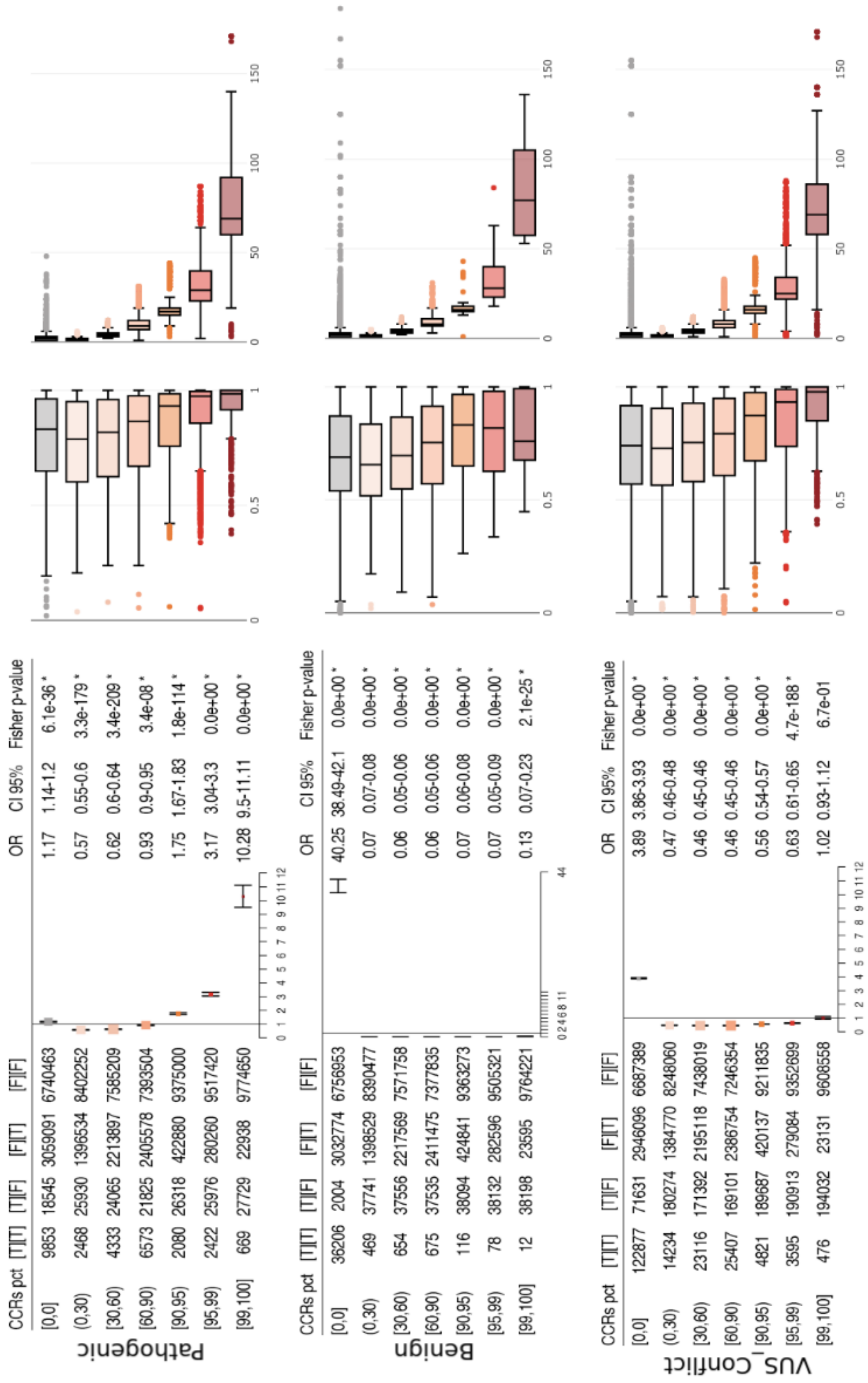

Pathogenic

CCRs pct	[T][T]	[T][F]	[F][T]	[F][F]	OR	CI 95%	Fisher p-value
[0,0]	9853	18545	3059091	6740463	1.17	1.14-1.2	6.1e-36 *
(0,30)	2468	25930	1396534	8402252	0.57	0.55-0.6	3.3e-179 *
[30,60)	4333	24065	2213897	7585209	0.62	0.6-0.64	3.4e-209 *
[60,90)	6573	21825	2405578	7393504	0.93	0.9-0.95	3.4e-08 *
[90,95)	2080	26318	422880	9375000	1.75	1.67-1.83	1.8e-114 *
[95,99)	2422	25976	280260	9517420	3.17	3.04-3.3	0.0e+00 *
[99,100]	669	27729	22938	9774650	10.28	9.5-11.11	0.0e+00 *

Benign

CCRs pct	[T][T]	[T][F]	[F][T]	[F][F]	OR	CI 95%	Fisher p-value
[0,0]	36206	2004	3032774	6756953	40.25	38.49-42.1	0.0e+00 *
(0,30)	469	37741	1398529	8390477	0.07	0.07-0.08	0.0e+00 *
[30,60)	654	37556	2217569	7571758	0.06	0.05-0.06	0.0e+00 *
[60,90)	675	37535	2411475	7377835	0.06	0.05-0.06	0.0e+00 *
[90,95)	116	38094	424841	9363273	0.07	0.06-0.08	0.0e+00 *
[95,99)	78	38132	282596	9505321	0.07	0.05-0.09	0.0e+00 *
[99,100]	12	38198	23595	9764221	0.13	0.07-0.23	2.1e-25 *

VUS_Conflict

CCRs pct	[T][T]	[T][F]	[F][T]	[F][F]	OR	CI 95%	Fisher p-value
[0,0]	122877	71631	2946096	6687389	3.89	3.86-3.93	0.0e+00 *
(0,30)	14234	180274	1384770	8248060	0.47	0.46-0.48	0.0e+00 *
[30,60)	23116	171392	2195118	7438019	0.46	0.45-0.46	0.0e+00 *
[60,90)	25407	169101	2386754	7246354	0.46	0.45-0.46	0.0e+00 *
[90,95)	4821	189687	420137	9211835	0.56	0.54-0.57	0.0e+00 *
[95,99)	3595	190913	279084	9352699	0.63	0.61-0.65	4.7e-188 *
[99,100]	476	194032	23131	9608558	1.02	0.93-1.12	6.7e-01

Supplementary figure 6: Comparison of OR tests, conservation and length of regions by group of ClinVar

variants. For each category of ClinVar missense, the plots show contingency tables with the sites counts, OR, 95% CI and p-values along with boxplots of distribution of CCRs regions length and interspecies amino acid conservation. Stars (*) highlight statistically significant ORs (p-value ≤ 0.05)

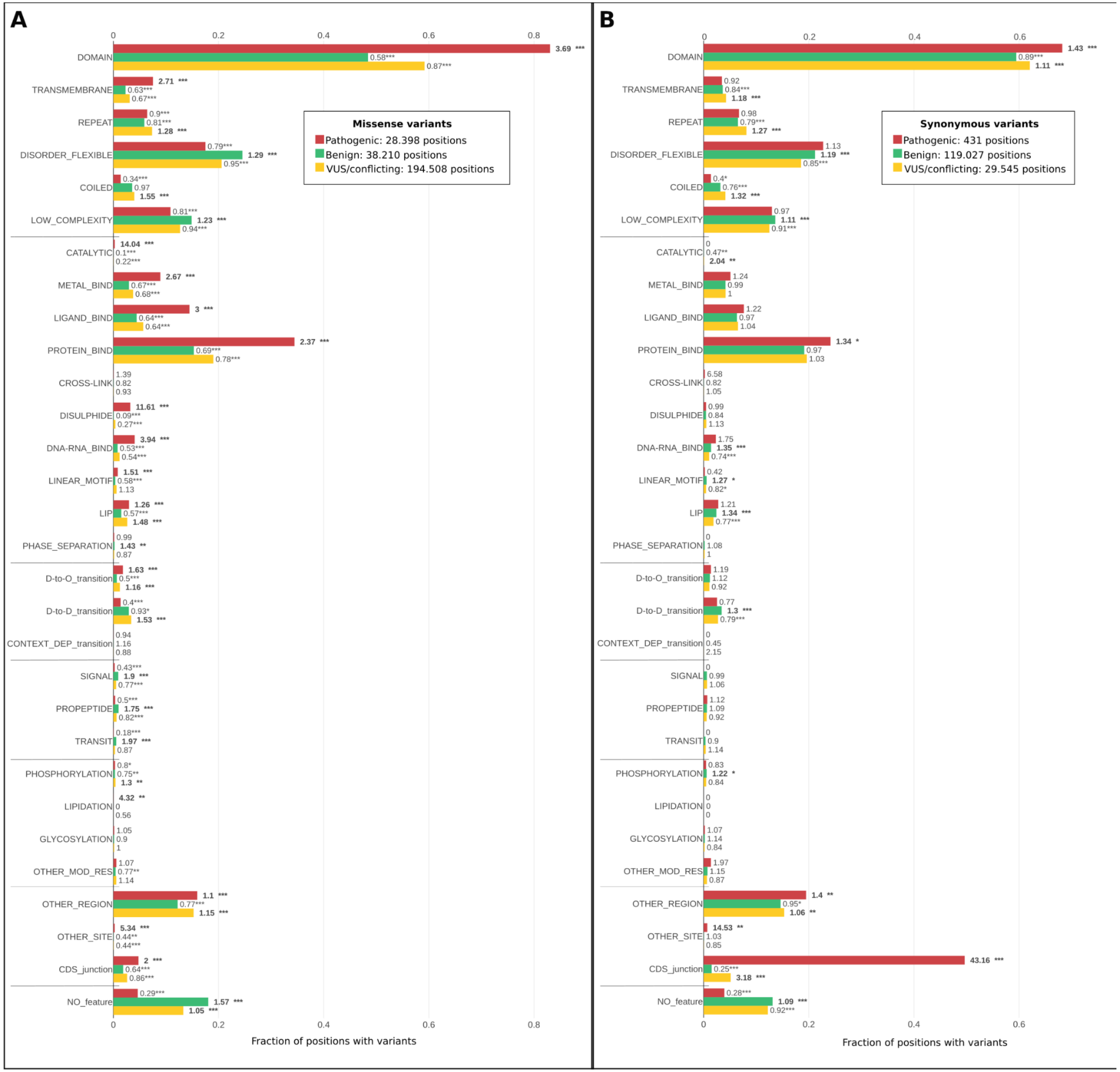
A
B
Missense variants
Pathogenic: 28.398 positions
Benign: 38.210 positions
VUS/conflicting: 194.508 positions
Synonymous variants
Pathogenic: 431 positions
Benign: 119.027 positions
VUS/conflicting: 29.545 positions
DOMAIN
TRANSMEMBRANE
REPEAT
DISORDER_FLEXIBLE
COILED
LOW_COMPLEXITY
CATALYTIC
METAL_BIND
LIGAND_BIND
PROTEIN_BIND
CROSS-LINK
DISULPHIDE
DNA-RNA_BIND
LINEAR_MOTIF
LIP
PHASE_SEPARATION
D-to-O_transition
D-to-D_transition
CONTEXT_DEP_transition
SIGNAL
PROPEPTIDE
TRANSIT
PHOSPHORYLATION
LIPIDATION
GLYCOSYLATION
OTHER_MOD_RES
OTHER_REGION
OTHER_SITE
CDS_junction
NO_feature
Fraction of positions with variants

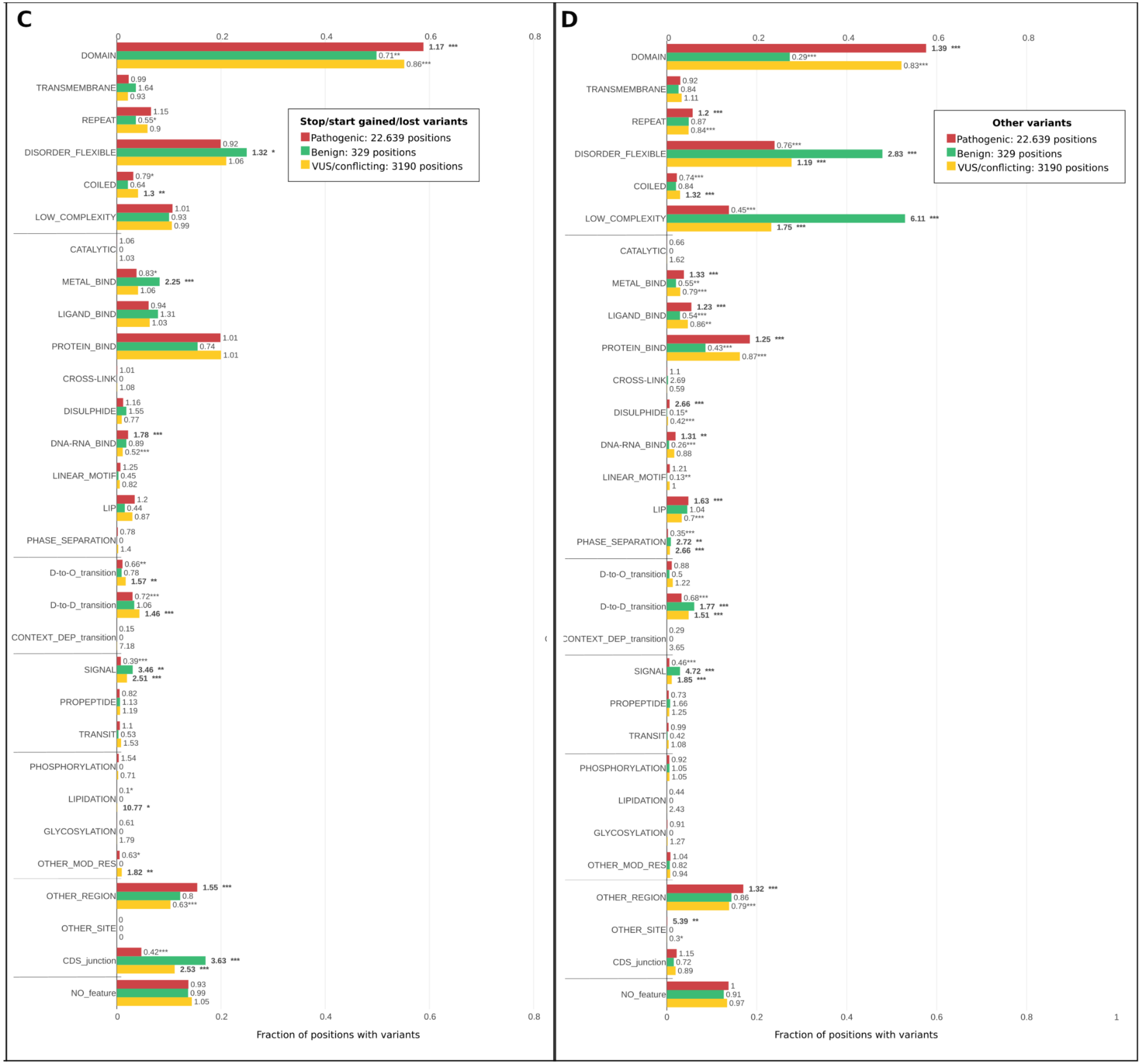
C
Stop/start gained/lost variants
Pathogenic: 22.639 positions
Benign: 329 positions
VUS/conflicting: 3190 positions
Fraction of positions with variants
D
Other variants
Pathogenic: 22.639 positions
Benign: 329 positions
VUS/conflicting: 3190 positions
Fraction of positions with variants

Supplementary Figure 7: Amino acid positions affected with clinically interpreted variants assessed by their location in the different protein features or functional sites. Each bar represents the fraction from the total positions with a feature that present variants assigned in ClinVar as having any of three types: Pathogenic/likely_pathogenic, Benign/Likely_benign or variant of uncertain significance or with conflicting interpretations of pathogenicity (VUS/conflicting), for four groups of variants: A) missense, B) synonymous, C) stop/start gained/lost and D) other variants.
The numbers next to the bars are the OR, calculated with a 95% CI, and represent the odds that protein positions in a particular feature/functional site will co-occur in combination with one of the three types of missense variants, compared to the odds of having such feature/functional site but with any of the other two types of missense of variants (See Supplementary Figure 6 for further information on how the contingency tables were constructed). Stars denote the statistical significance (p-value) according to a two-tailed Fisher's exact test, the more stars the higher the significance (no star, not significant: $p\text{-value} > 0.05$; *: $0.05 \geq p\text{-value} > 0.01$; **: $0.01 \geq p\text{-value} > 0.001$; ***: $p\text{-value} \leq 0.001$). Cases where OR>1 and 95% CI>1 with $p\text{-value} \leq 0.05$ are bolded, and highlight when there is a positive association between the category of protein feature/functional site and a type of missense variants.
