# Supplementary material for: Mapping the Constrained Coding Regions in the Human Genome to Their Corresponding Proteins
Source: J Mol Biol. 2023 Jan 30;435(2):167892. doi: 10.1016/j.jmb.2022.167892 (PMC9875310; doi:10.1016/j.jmb.2022.167892)
Supplement: Supplementary Methods [file mmc8.pdf]

Mapping the Constrained Coding Regions in the human genome to their corresponding proteins

Marcia A. Hasenahuer[1,2,5], Alba Sanchis-Juan[3,4,6], Roman A. Laskowski[1], James A. Baker[1], James D. Stephenson[1], Christine A. Orengo[5], F. Lucy Raymond[2,4], Janet M. Thornton[1]

Supplementary Methods

Constraint metrics

The variability constraint metric scores we employed in the present work were downloaded from gnomAD2.1.1 (see Supplementary Methods Table 1 for specific files). These metrics are available by transcript and by gene (i.e. Ensembl canonical transcript) and estimate overall intolerance (level of constraint) of the transcript or gene to certain types of variation: missense, synonymous and loss-of-function (LoF) including nonsense (stop-gained), frameshift, splice acceptor, and splice donor variants, all caused by single nucleotide changes.

These metrics are essentially based on comparing the observed variant counts *(o)* (from gnomAD2.1.1) to an expectation of variability *(e)* for a gene or transcript. *o and e* are the starting point from which the constraint metrics are derived: I) o/e ratio; II) OEUF, the upper bound fraction of a 90% confidence interval around the o/e ratio; III) pLI, the probability of a gene/transcript to be loss-of-function intolerant in heterozygosity; and IV) Z-score: a signed score of the deviation of *o* from *e.*

For the analysis in present work, we specifically employed gene-based pLI and missense OEUF scores. In order to provide a basic framework for understanding the principles and interpretation of these scores, in the following subsections we briefly describe the fundamentals of how *o* and *e* are estimated and how the different scores are derived from them. However, for further information about these metrics, refer to the the flagship

publications of ExAC [1] (more in detail in the Supplementary Information section 4, https://static-content.springer.com/esm/art%3A10.1038%2Fnature19057/MediaObjects/41586_2016_BFnature19057_MOESM240_ESM.pdf) and gnomAD2 [2].

I) o/e ratio:

- *o*, the observed variant count, is the number of unique single nucleotide variants in the transcript of each gene that were found at a frequency of <0.1%, passed all filters and were at sites with a median sequencing depth ≥1. The counts represent the number of unique variants and not the allele count of these variants.
- *e*, the expected variants count, were obtained by estimating a depth corrected probability of mutation for each gene, taking into account sequence context, coverage of sequencing and methylation.

When a gene has a low o/e value, it is under stronger selection for that class of variation than a gene with a higher value.

II) OEUFs:

Because variant counts depend on gene size and sample size, the precision of the *o/e* values varies a lot from gene to gene. Therefore in addition to the *o/e* value, gnomAD provides the 90% confidence interval (CI) for each of the *o/e* values. It is advised to use an OEUF < 0.35 as a more conservative threshold for saying that a gene/transcript is constrained for a type of variant, and hence we adopt this threshold in the present work. OEUFs are available for LoF (‘LOEUF’), missense and synonymous variants.

III) pLI:

Gives the probability of being a haploinsufficient gene (i.e. loss of a single copy of the gene is not tolerated). pLI was developed using the expectation-maximization (EM) algorithm, where three categories were defined to classify genes with respect to sensitivity to LoF variants: null (where LoF - heterozygous or homozygous- is completely tolerated by natural selection, i.e. loss of both copies of the gene is accepted), recessive (where heterozygous LoF variants are tolerated but homozygous are not), and haploinsufficient (where heterozygous LoF is not tolerated, i.e both copies of the gene must be functional). Then, observed and expected LoF variant counts were used to determine pLI, which is the probability that a given transcript falls into the third category. A threshold of pLI ≥ 0.9 can be used to get a set of “essential” transcripts/genes which are extremely intolerant to LoF variants.

IV) Z-scores:

Z-scores are based on Chi-squared values for the deviation of observed from expected variants (for synonymous and missense). Higher (more positive) Z-scores indicate that the gene/transcript is more intolerant for the type of variation (missense or synonymous), and the contrary is interpreted for Z-scores under 0.

Aggregation of protein features annotations and clinically interpreted variants

I. Protein features, functional annotations and conservation score (Figure 9, left blue-green panel) were obtained from the different databases listed in Supplementary Methods Table 1, following the criteria described in Supplementary Methods Table 2.

II. Clinically interpreted missense variants (Figure 9, middle yellow panel) were obtained from ClinVar (see Supplementary Methods Table 1). We annotated the VCF file with Ensembl VEP v101 and restricted the dataset to those variants annotated as pathogenic/likely_pathogenic, benign/likely_benign, and combined the variants of uncertain

significance (VUS) with those having conflicting interpretations of pathogenicity into a single subset named ‘VUS_conflictive’.

Resource/Database	Description/specific datasets	Version/date of accession
Ensembl [3], https://www.ensembl.org/index.html	GTF files: http://ftp.ensembl.org/pub/release-101/gtf/homo_sapiens/	v101
gnomAD [2], https://gnomad.broadinstitute.org/	**Variants:** VCF and coverage files were obtained from gs://gcp-public-data--gnomad/release/3.0 using *gsutil* tool (https://cloud.google.com/storage/docs/gsutil)	v3.0
	**Constraint metrics:** pLI, LOEUF, missense OEUF, Z-scores we obtained<br>• *by gene:* https://storage.googleapis.com/gcp-public-data--gnomad/release/2.1.1/constraint/gnomad.v2.1.1.lof_metrics.by_gene.txt.bgz,<br>• *by transcript:* https://storage.googleapis.com/gcp-public-data--gnomad/release/2.1.1/constraint/gnomad.v2.1.1.lof_metrics.by_transcript.txt.bgz	v2.1.1
UniProtKB [4], https://www.uniprot.org/	Protein features obtained via REST API: https://www.ebi.ac.uk/proteins/api/doc/index.html#/features	March 2021
VarSite [5], https://www.ebi.ac.uk/thornton-srv/databases/cgi-bin/VarSite/GetPage.pl?home=TRUE	Data provided by Roman A. Laskowski. VarSite gathers structural annotations via PDBsum [6] using also structures from homologous proteins.	March 2021
M-CSA, [7] https://www.ebi.ac.uk/thornton-srv/m-csa/	List of catalytic residues: https://www.ebi.ac.uk/thornton-srv/m-csa/api/residues/?format=json	June 2021
BioLip [8], database for	List of artifact ligands:	April 2021

biologically relevant ligand-protein binding interactions, https://zhanggroup.org/BioLiP/	https://zhanggroup.org/BioLiP/ligand_list	
MobiDB [9], database for disorder and mobility annotations, https://mobidb.bio.unipd.it/	Features obtained via REST API, using the MobiDB vocabulary: https://mobidb.bio.unipd.it/about/vocabulary	April 2021
ELM [10], The Eukaryotic Linear Motif resource, http://elm.eu.org/searchdb.html	All instances, filtered for true positives and Human: http://elm.eu.org/instances.html?q=*	February 2021
ClinVar [11], NCBI public archive for clinically interpreted variants, https://www.ncbi.nlm.nih.gov/clinvar/	VCF file: https://ftp.ncbi.nlm.nih.gov/pub/clinvar/vcf_GRCh38/archive_2.0/2021/clinvar_20210814.vcf.gz	August 2021

Supplementary Methods Table 1: list of databases and resources employed in the present work

Feature	Description	Databases and criteria
DOMAIN	Protein domain	UniProtKB: feature 'DOMAIN', and/or VarSite: presence of a CATH [12] domain
REPEAT	Repeated sequence motif or domain	UniProtKB: feature 'REPEAT'
TRANSMEMBRANE	Transmembrane region	UniProtKB: feature 'TRANSMEM'
COILED	Coiled-coil region	UniProtKB: feature 'COILED'
LOW_COMPLEXITY	Low-complexity region, in terms of amino acid composition	UniProtKB: feature 'COMPBIAS', and/or MobiDB: feature 'prediction_low_complexity_merge'
CATALYTIC	Catalytic site	M-CSA: if assigned a role in catalysis, and/or UniProtKB: feature 'ACT_SITE'
METAL_BIND	Interaction with a metal ion	UniProtKB: any of 'METAL' or 'ZN_FING', and/or VarSite annotations
LIGAND_BIND	Interaction with a small molecule	UniProtKB: any of 'NP_BIND' or 'BINDING', and/or annotated in VarSite and filtering out artifact ligands from BioLip
PROTEIN_BIND	Interaction with another protein	VarSite

CROSS_LINK	A protein cross-linking interaction	UniProtKB: feature 'CROSSLNK'
DISULPHIDE	Disulfide (S-S) bond	UniProtKB: feature 'DISULFID', and/or VarSite
DNA_RNA_BIND	Interaction with DNA or RNA	UniProtKB: feature 'DNA_BIND' and/or VarSite
LINEAR_MOTIF	Linear motif	UniProtKB: feature 'MOTIF', and/or ELM: true positives for Human
LIP	Linear interacting peptide	MobiDB: any of 'curated_lip_merge', 'derived_lip_merge' or 'prediction_lip_merge'
DISORDER_MOBILE	Instrinsically disordered or highly mobile region	MobiDB: we considered as disordered/highly mobile those protein sites annotated with any of the following features:<br>• *Disordered:* 'curated_disorder_merge', 'homology_disorder_merge', 'prediction_disorder_mobidb_lite' or 'prediction_disorder_th_50'<br>• *Missing coordinates* in experimentally determined structures: 'derived_missing_residues_context_dependent_th_90' or 'derived_missing_residues_th_90'<br>• *Highly mobile in NMR structures*: 'derived_mobile_context_dependent_th_90' or 'derived_mobile_th_90'
D-to-D_TRANSITION	A region that remains disordered upon binding with a protein partner	MobiDB: assigned with any of 'curated_binding_mode_disorder_to_disorder_merge' or 'derived_binding_mode_disorder_to_disorder_priority'
D-to-O_TRANSITION	A region that transitions from disordered to ordered upon binding with a protein partner	MobiDB: assigned with 'derived_binding_mode_disorder_to_order_priority'
CONTEXT_DEP_TRANSITION	A region that has alternative binding configurations, which change with the cellular conditions and different partners	MobiDB: assigned with 'derived_binding_mode_context_dependent_priority'
PHASE_SEPARATION	A region that drives liquid-liquid phase separation	MobiDB: assigned with: 'curated_phase_separation_merge' or

	(LLPS)	'homology_phase_separation_merge'. LLPS annotations are gathered from PhaSePro [13]
SIGNAL	Signal peptide	UniProtKB: feature 'SIGNAL'
PROPEP	Propeptide region (cleaved during protein "maturation")	UniProtKB: feature 'PROPEP'
TRANSIT	Region necessary for the transport of a protein encoded by a nuclear gene to a particular organelle (e.g. peroxisome, mitochondrion)	UniProtKB: feature 'TRANSIT'
PHOSPHORYLATION	A Ser, Thr or Tyr post-translationally modified with phosphorylation	UniProtKB: feature 'MOD_RES' with presence of 'Phospho' or 'phospho' in description
LIPIDATION	A site that acquires covalently attached lipid group(s)	UniProtKB: feature 'LIPID'
GLYCOSYLATION	A glycosylated site (covalently attached glycan group (mono-, di-, or polysaccharide))	UniProtKB: feature 'CARBOHYD'
PTM_OTHER	Other post-translational modifications, excluding phosphorylation: methylation, acetylation, amidation, formation of pyrrolidone carboxylic acid, isomerization, hydroxylation, sulfation, flavin-binding, cysteine oxidation and nitrosylation	UniProtKB: feature 'MOD_RES' without presence of 'Phospho' or 'phospho' in description
OTHER_REGION	A region of interest that cannot be described in other subsections of UniProtKB	UniProtKB: feature 'REGION'
OTHER_SITE	A site of interest that cannot be described in other subsections of UniProtKB	UniProtKB: feature 'SITE'
CDSjunction	Protein positions encoded by bases in CDS (CoDing Sequence) junctions	Ensembl: coding bases in exon-intron boundaries were translated to protein, using the same strategy as described in the “Mapping the CCRs to protein amino acids” section in Methods
NO_feature	Having none of the previous 29 features	-

CONSERVATION	Inter-species conservation score	Obtained from VarSite, which uses Blastp [14] to search for homologous sequences in UniProtKB, and then ScoreCons [15] for computing the conservation scores

Supplementary Methods Table 2: Databases and criteria employed for obtaining by-residue protein features annotations and conservation score.

Odds ratios tests for enrichment

To calculate the ORs, we compare the observed frequency with the enrichment of such CCRpct intervals with all other protein feature annotations. To do so, a 2x2 contingency table was constructed for each combination of feature annotation and CCRpct interval with the following cells: (a) the count of all residues at the CCRpct group that intersected the given feature annotation, (b) the count of all residues outside the CCRpct group that intersected the given feature annotation, (c) the count of all residues at the CCRpct group that intersect with other different feature annotations, and (d) the count of all residues outside the CCRpct group that intersect with other different feature annotations. The two-tailed Fisher's and one-tailed exact tests (Fisher 1970) were used to estimate the P-value and 95% confidence interval (CI 95%) for the OR of each contingency table, by testing the null hypothesis of a uniform distribution of CCRpct groups intersecting protein features and using *fisher.test* function from the *stats* library in R.

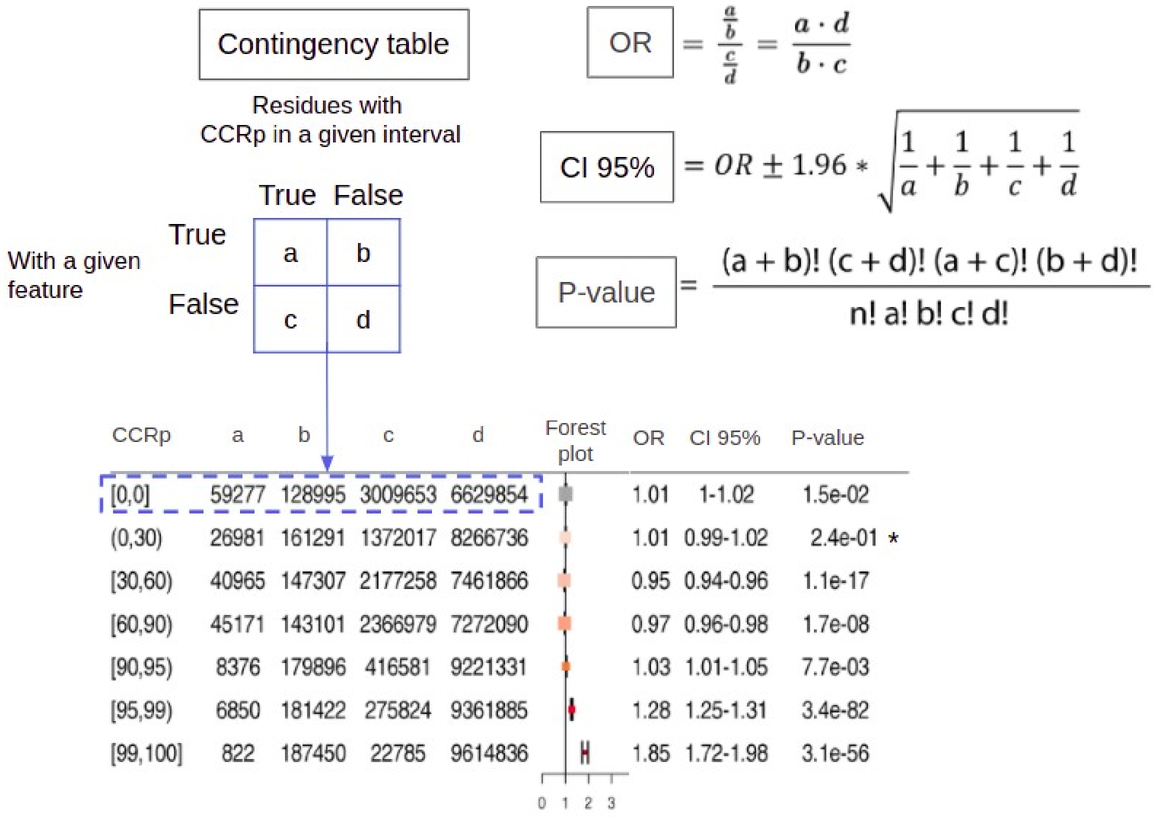

Supplementary Methods Figure 1: Example illustrating how 2x2 contingency tables were built for performing the odds ratios (OR) tests by counting residues presenting the co-ocurrence of the different CCRs percentiles groups with a protein feature, i.e. any of DOMAIN, CATALYTIC, DISORDER_MOBILE, etc or in NO_FEATURE for absence of any annotation or with the presence of PATHOGENIC, BENIGNG or VUS_conflict missense variants. From the OR test, three scenarios are possible: (1) if OR > 1.0 along with the CI 95% and P-value<=0.05: the residues with the given feature are OR times more likely associated to the given CCRp group, (2) OR < 1.0 along with the 95% CI and p-value<=0.05: the residues with the given feature are OR times less likely to have the given CCRp, and (3) the 95% confidence interval crosses over OR =1.0 and p-value>0.05, null hypothesis cannot be rejected hence no significant association is observed. A star (*) highlights this last situation.

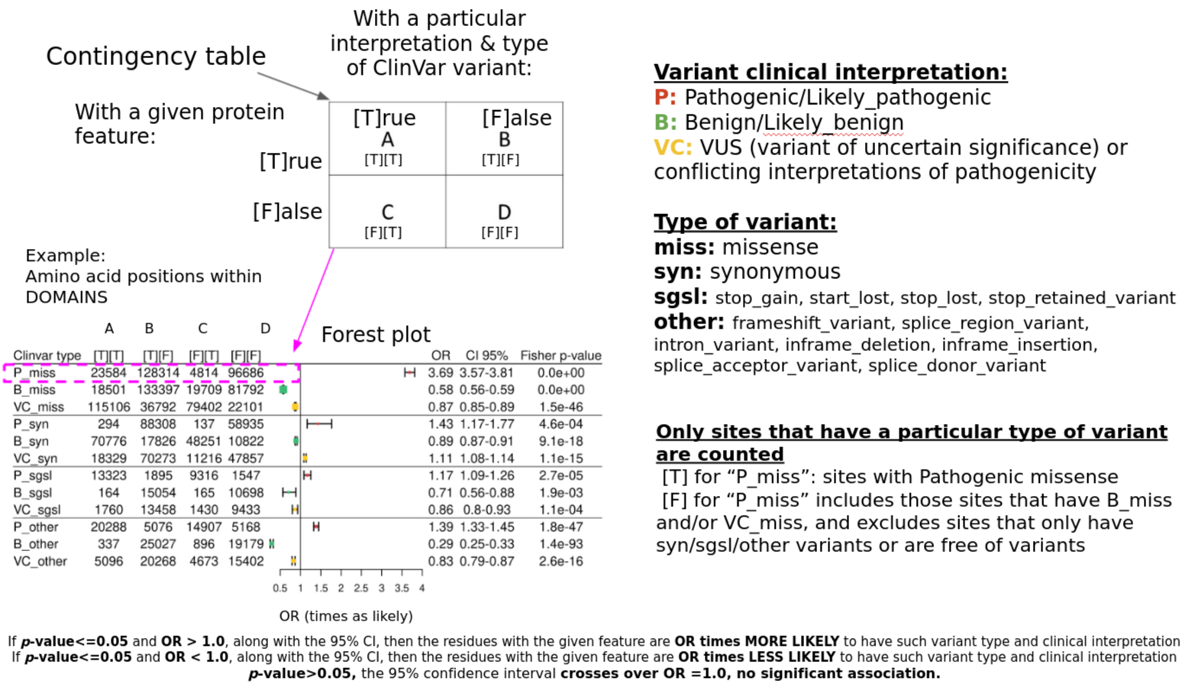

Supplementary Methods Figure 2: Example illustrating how contingency tables were built for performing the odds ratios (OR) tests, comparing each protein feature co-occurrence with different types of clinically interpreted variants from ClinVar.

References

[1] M. Lek, K.J. Karczewski, E.V. Minikel, K.E. Samocha, E. Banks, T. Fennell, A.H. O'Donnell-Luria, J.S. Ware, A.J. Hill, B.B. Cummings, T. Tukiainen, D.P. Birnbaum, J.A. Kosmicki, L.E. Duncan, K. Estrada, F. Zhao, J. Zou, E. Pierce-Hoffman, J. Berghout, D.N. Cooper, N. Deflaux, M. DePristo, R. Do, J. Flannick, M. Fromer, L. Gauthier, J. Goldstein, N. Gupta, D. Howrigan, A. Kiezun, M.I. Kurki, A.L. Moonshine, P. Natarajan, L. Orozco, G.M. Peloso, R. Poplin, M.A. Rivas, V. Ruano-Rubio, S.A. Rose, D.M. Ruderfer, K. Shakir, P.D. Stenson, C. Stevens, B.P. Thomas, G. Tiao, M.T. Tusie-Luna, B. Weisburd, H.-H. Won, D. Yu, D.M. Altshuler, D. Ardissino, M. Boehnke, J. Danesh, S. Donnelly, R. Elosua, J.C. Florez, S.B. Gabriel, G. Getz, S.J. Glatt, C.M. Hultman, S. Kathiresan, M. Laakso, S. McCarroll, M.I. McCarthy, D. McGovern, R. McPherson, B.M. Neale, A. Palotie, S.M. Purcell, D. Saleheen, J.M. Scharf, P. Sklar, P.F. Sullivan, J. Tuomilehto, M.T. Tsuang, H.C. Watkins, J.G. Wilson, M.J. Daly, D.G. MacArthur, Exome Aggregation Consortium, Analysis of protein-coding genetic variation in 60,706 humans, Nature. 536 (2016) 285–291.

[2] K.J. Karczewski, L.C. Francioli, G. Tiao, B.B. Cummings, J. Alföldi, Q. Wang, R.L. Collins, K.M. Laricchia, A. Ganna, D.P. Birnbaum, L.D. Gauthier, H. Brand, M. Solomonson, N.A. Watts, D. Rhodes, M. Singer-Berk, E.M. England, E.G. Seaby, J.A. Kosmicki, R.K. Walters, K. Tashman, Y. Farjoun, E. Banks, T. Poterba, A. Wang, C. Seed, N. Whiffin, J.X. Chong, K.E. Samocha, E. Pierce-Hoffman, Z. Zappala, A.H. O'Donnell-Luria, E.V. Minikel, B. Weisburd, M. Lek, J.S. Ware, C. Vittal, I.M. Armean, L. Bergelson, K. Cibulskis, K.M. Connolly, M. Covarrubias, S. Donnelly, S. Ferriera, S. Gabriel, J. Gentry, N. Gupta, T. Jeandet, D. Kaplan, C. Llanwarne, R. Munshi, S. Novod, N. Petrillo, D. Roazen, V. Ruano-

Rubio, A. Saltzman, M. Schleicher, J. Soto, K. Tibbetts, C. Tolonen, G. Wade, M.E. Talkowski, Genome Aggregation Database Consortium, B.M. Neale, M.J. Daly, D.G. MacArthur, The mutational constraint spectrum quantified from variation in 141,456 humans, Nature. 581 (2020) 434–443.

[3] K.L. Howe, P. Achuthan, J. Allen, J. Allen, J. Alvarez-Jarreta, M.R. Amode, I.M. Armean, A.G. Azov, R. Bennett, J. Bhai, K. Billis, S. Boddu, M. Charkhchi, C. Cummins, L. Da Rin Fioretto, C. Davidson, K. Dodiya, B. El Houdaigui, R. Fatima, A. Gall, C. Garcia Giron, T. Grego, C. Guijarro-Clarke, L. Haggerty, A. Hemrom, T. Hourlier, O.G. Izuogu, T. Juettemann, V. Kaikala, M. Kay, I. Lavidas, T. Le, D. Lemos, J. Gonzalez Martinez, J.C. Marugán, T. Maurel, A.C. McMahon, S. Mohanan, B. Moore, M. Muffato, D.N. Oheh, D. Paraschas, A. Parker, A. Parton, I. Prosovetskaia, M.P. Sakthivel, A.I.A. Salam, B.M. Schmitt, H. Schuilenburg, D. Sheppard, E. Steed, M. Szpak, M. Szuba, K. Taylor, A. Thormann, G. Threadgold, B. Walts, A. Winterbottom, M. Chakiachvili, A. Chaubal, N. De Silva, B. Flint, A. Frankish, S.E. Hunt, G.R. IIsley, N. Langridge, J.E. Loveland, F.J. Martin, J.M. Mudge, J. Morales, E. Perry, M. Ruffier, J. Tate, D. Thybert, S.J. Trevanion, F. Cunningham, A.D. Yates, D.R. Zerbino, P. Flicek, Ensembl 2021, Nucleic Acids Res. 49 (2021) D884–D891.

[4] UniProt Consortium, UniProt: the universal protein knowledgebase in 2021, Nucleic Acids Res. 49 (2021) D480–D489.

[5] R.A. Laskowski, J.D. Stephenson, I. Sillitoe, C.A. Orengo, J.M. Thornton, VarSite: Disease variants and protein structure, Protein Sci. 29 (2020) 111–119.

[6] R.A. Laskowski, J. Jabłońska, L. Pravda, R.S. Vařeková, J.M. Thornton, PDBsum: Structural summaries of PDB entries, Protein Sci. 27 (2018) 129–134.

[7] A.J.M. Ribeiro, G.L. Holliday, N. Furnham, J.D. Tyzack, K. Ferris, J.M. Thornton, Mechanism and Catalytic Site Atlas (M-CSA): a database of enzyme reaction mechanisms and active sites, Nucleic Acids Res. 46 (2018) D618–D623.

[8] J. Yang, A. Roy, Y. Zhang, BioLiP: a semi-manually curated database for biologically relevant ligand-protein interactions, Nucleic Acids Res. 41 (2013) D1096–103.

[9] D. Piovesan, M. Necci, N. Escobedo, A.M. Monzon, A. Hatos, I. Mičetić, F. Quaglia, L. Paladin, P. Ramasamy, Z. Dosztányi, W.F. Vranken, N.E. Davey, G. Parisi, M. Fuxreiter, S.C.E. Tosatto, MobiDB: intrinsically disordered proteins in 2021, Nucleic Acids Res. 49 (2021) D361–D367.

[10] M. Kumar, M. Gouw, S. Michael, H. Sámano-Sánchez, R. Pancsa, J. Glavina, A. Diakogianni, J.A. Valverde, D. Bukirova, J. Čalyševa, N. Palopoli, N.E. Davey, L.B. Chemes, T.J. Gibson, ELM-the eukaryotic linear motif resource in 2020, Nucleic Acids Res. 48 (2020) D296–D306.

[11] M.J. Landrum, J.M. Lee, M. Benson, G.R. Brown, C. Chao, S. Chitipiralla, B. Gu, J. Hart, D. Hoffman, W. Jang, K. Karapetyan, K. Katz, C. Liu, Z. Maddipatla, A. Malheiro, K. McDaniel, M. Ovetsky, G. Riley, G. Zhou, J.B. Holmes, B.L. Kattman, D.R. Maglott, ClinVar: improving access to variant interpretations and supporting evidence, Nucleic Acids Res. 46 (2018) D1062–D1067.

[12] I. Sillitoe, N. Bordin, N. Dawson, V.P. Waman, P. Ashford, H.M. Scholes, C.S.M. Pang, L. Woodridge, C. Rauer, N. Sen, M. Abbasian, S. Le Cornu, S.D. Lam, K. Berka, I.H. Varekova, R. Svobodova, J. Lees, C.A. Orengo, CATH: increased structural coverage of functional space, Nucleic Acids Res. 49 (2021) D266–D273.

[13] B. Mészáros, G. Erdős, B. Szabó, É. Schád, Á. Tantos, R. Abukhairan, T. Horváth, N. Murvai, O.P. Kovács, M. Kovács, S.C.E. Tosatto, P. Tompa, Z. Dosztányi, R. Pancsa,

PhaSePro: the database of proteins driving liquid-liquid phase separation, Nucleic Acids Res. 48 (2020) D360–D367.
[14] S.F. Altschul, W. Gish, W. Miller, E.W. Myers, D.J. Lipman, Basic local alignment search tool, J. Mol. Biol. 215 (1990) 403–410.
[15] W.S.J. Valdar, Scoring residue conservation, Proteins. 48 (2002) 227–241.
[16] W. McLaren, L. Gil, S.E. Hunt, H.S. Riat, G.R.S. Ritchie, A. Thormann, P. Flicek, F. Cunningham, The Ensembl Variant Effect Predictor, Genome Biol. 17 (2016) 122.
[17] F. Quaglia, B. Mészáros, E. Salladini, A. Hatos, R. Pancsa, L.B. Chemes, M. Pajkos, T. Lazar, S. Peña-Díaz, J. Santos, V. Ács, N. Farahi, E. Fichó, M.C. Aspromonte, C. Bassot, A. Chasapi, N.E. Davey, R. Davidović, L. Dobson, A. Elofsson, G. Erdős, P. Gaudet, M. Giglio, J. Glavina, J. Iserte, V. Iglesias, Z. Kálmán, M. Lambrughi, E. Leonardi, S. Longhi, S. Macedo-Ribeiro, E. Maiani, J. Marchetti, C. Marino-Buslje, A. Mészáros, A.M. Monzon, G. Minervini, S. Nadendla, J.F. Nilsson, M. Novotný, C.A. Ouzounis, N. Palopoli, E. Papaleo, P.J.B. Pereira, G. Pozzati, V.J. Promponas, J. Pujols, A.C.S. Rocha, M. Salas, L.R. Sawicki, E. Schad, A. Shenoy, T. Szaniszló, K.D. Tsirigos, N. Veljkovic, G. Parisi, S. Ventura, Z. Dosztányi, P. Tompa, S.C.E. Tosatto, D. Piovesan, DisProt in 2022: improved quality and accessibility of protein intrinsic disorder annotation, Nucleic Acids Res. 50 (2022) D480–D487.
[18] S.R.A. Fisher, Statistical Methods for Research Workers, Oliver and Boyd, 1970.
